# A retrospective study of the MDS criteria for prodromal Parkinson’s disease in the general population

**DOI:** 10.1038/s41531-024-00739-6

**Published:** 2024-06-26

**Authors:** Gijs W. de Klerk, Teus van Laar, Sanne K. Meles

**Affiliations:** Department of Neurology, University of Groningen, University Medical Center Groningen, Groningen, The Netherlands

**Keywords:** Parkinson's disease, Risk factors, Signs and symptoms

## Abstract

The Movement Disorder Society developed research criteria for the detection of the prodromal phase of Parkinson’s disease (PD). Accurate identification of this phase is essential for early interventions. Therefore, we investigated the diagnostic value of these research criteria in the general population. Lifelines is an ongoing cohort study of 167,000 participants from the general population of the Northern Netherlands. 160 participants self-reported to have developed PD during three rounds of follow-up of five years each. Data were available to infer six out of eleven risk markers, and six out of twelve prodromal markers. We retrospectively compared the criteria in the prodromal stage of a group of 160 ‘converters’ with 320 age- and sex-matched controls. The overall incidence rate of PD was 0.20 per 1.000 person-years (95% CI: 0.049−0.36), increasing with age and rates were higher in men. The median probability for prodromal PD in PD-converters was 1.29% (interquartile range: 0.46−2.9), compared to 0.83% (0.39−1.8) for controls (*P* = 0.014). The MDS set of criteria for prodromal PD had an ROC-AUC of 0.577, and was therefore not sufficient to adequately predict conversion to PD. We were unable to predict conversion to PD in the general population using a selection of the prodromal PD research criteria. Ancillary investigations are required to improve the diagnostic accuracy of the criteria, but most are precluded from large-scale use. Strategies, including olfactory tests or alpha-synuclein seeding amplification assays may improve the detection of prodromal PD in the general population.

## Introduction

Parkinson’s disease (PD) is a clinical diagnosis, which relies on the presence of specific motor symptoms: bradykinesia, rigidity and/or tremor^1^. Prior to the development of these typical motor symptoms, a prodromal phase can be recognized in approximately one-third of all PD patients^2^. During this phase, a person experiences a variety of non-motor symptoms, such as autonomic symptoms or sleep disorders. Compared to clinically established PD, neurodegeneration in the prodromal phase is relatively limited. This makes prodromal subjects prime candidates for clinical trials testing disease-modifying compounds^3^. Identifying the prodromal phase reliably is crucial as disease-modifying treatments are currently in development^4^.

The Movement Disorder Society (MDS) has developed criteria to identify prodromal PD^5,6^. These criteria can be used to determine the probability of prodromal PD in an individual by combining the age-based risk with specific risk and prodromal markers. If the calculated probability exceeds 80%, the individual can be considered to have ‘probable prodromal PD’. These criteria encompass factors easily assessed through questionnaires, such as excessive daytime sleepiness or constipation, as well as markers requiring ancillary investigations. For example, suspected REM sleep behavior disorder (RBD) should be confirmed with a video-polysomnography (PSG). Markers vary in their associated likelihood ratios (LR), with constipation at 2.5, depression at 1.6, and markers like orthostatic hypotension or PSG-proven RBD at 18.5 and 130, respectively.

Several studies have investigated the validity of the MDS research criteria in both general and enriched populations. Participants in enriched cohorts, selected on features associated with increased PD risk (e.g., RBD or genetic mutations), often met the 80% threshold, yielding high sensitivity rates of up to 80%^7–9^. In contrast, the criteria exhibit lower sensitivity in the general population, with the Bruneck study reporting 67%, and other cohorts showing even lower rates^10–13^. However, the criteria tend to have higher specificity in the general population, approaching 100%. Population-based studies performed so far have been relatively small, with only 12-22 incident PD cases (7-13). Further validation in larger population-based cohorts is needed.

The Lifelines biobank includes over 167,000 participants from the Northern Netherlands with clinical data and biosamples collected since 2006. Notably, participant inclusion was not dependent on predefined criteria; instead, individuals were invited to complete comprehensive questionnaires on various physical and mental health topics. Thus, the Lifelines biobank represents a valuable resource for the empirical validation of the MDS research criteria in the general population. Since its inception, 174 Lifelines participants self-reported a PD diagnosis. To the best of our knowledge, this is the largest cohort of incident PD cases derived from a population-based study.

In this retrospective study, we investigated whether individuals with prodromal PD could be reliably identified using a selection of risk and prodromal markers from the MDS research criteria, that were available from the Lifelines database. We conducted this analysis by comparing data from participants who reported being diagnosed with PD during Lifelines follow-up with an age- and sex-matched control group.

## Results

### Demographics

**Fig. 1:**
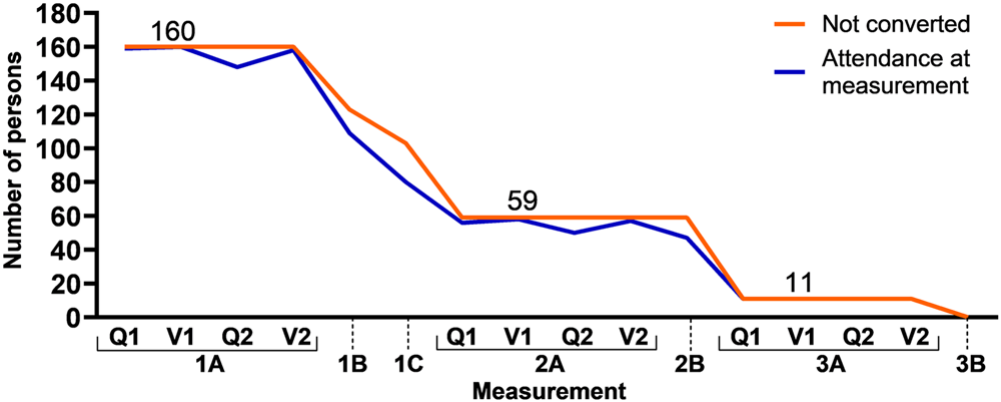
Timing of conversion and attendance at the assessments. 1 A, 2 A and 3 A are assessment rounds consisting of two questionnaires (Q) and two visits (V), each separated by five years. Between rounds, questionnaires (1B, 1 C, 2B, 3B) are sent out. The X-axis is not linear for time.

The Lifelines database included 174 subjects who self-reported a diagnosis of PD after their first assessment. Fourteen were excluded, resulting in a group of 160 converters, of which 63% were male (*n* = 100). The mean age at baseline (i.e., inclusion in Lifelines) was 59 ± 11 years. Participants reported a PD-diagnosis at a mean age of 64 ± 10 years. The mean duration of follow-up before reported conversion was 5.6 ± 4.0 years. Details for 160 converters and 320 age- and sex-matched controls are indicated in Supplementary table 1. Of the 160 converters, 101 had converted at the start of the second round of assessments. 48 of the remaining 59 had converted at the start of the third round of assessments, and the remaining 11 reported conversion at a later questionnaire (Fig. 1).

Of the 160 converters, a subgroup of 73 converters had near complete data (≤2 markers missing). 71% was male (*n* = 52) and the mean age at baseline was 57 ± 10 years. The mean duration of follow-up was 8.7 ± 3.1 years. Of this group, <10 converted in 1 C, <20 in 2 A, 45 in 3 A and 10 in 3B. Details for this subgroup and its matched control group are indicated in Supplementary table 2.

### The incidence of Parkinson’s disease in Lifelines

**Fig. 2:**
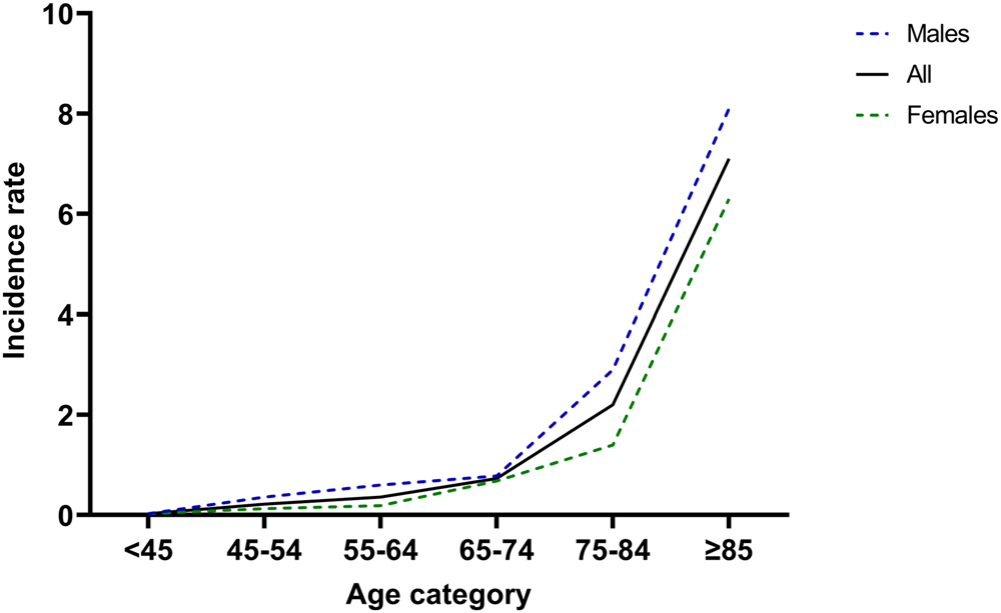
Incidence rate of Parkinson’s disease per age category. Incidence rate per 1000 person-years of Parkinson’s disease in the lifelines population per age category. Shown for males, females and all subjects combined.

The cohort consisted of 166,181 subjects. After excluding 29,770 cases, the remaining 136,411 subjects were used for calculating the incidence. The exclusions primarily consisted of cases assessed only once (*n* = 29,680), while a small minority were removed due to subjects reporting a stroke before the PD diagnosis, or a diagnosis of PD at first assessment. The 160 incident cases of PD were reported within a total of 785,962 person-years, resulting in an overall incidence rate of 0.20 (95% CI 0.17−0.24) per 1000 person-years. The incidence rate increased per age category, with individuals aged >85 years having the highest overall incidence rates (Fig. 2). Men had a higher incidence rate for PD than women. Supplementary table 3 shows the incidence rates per age category for the whole group, and for men and women separately.

### Risk markers, prodromal features and the MDS research criteria

Of the available risk markers, non-use of caffeine and a positive family history were more common in converters compared to controls (*P* = 0.004 and *P* < 0.001, respectively). In contrast, type 2 diabetes mellitus (T2DM) was more frequent in the controls (*P* = 0.018). Of the prodromal markers, only a positive answer to the surrogate question for possible RBD was more common in the converters (P = 0.006). The other prodromal markers were similar between the two groups (Supplementary Table 4*)*. As the control group was matched for age and sex, the pretest probability was similar between the groups *(*Supplementary Table 5*)*. The LR for risk and prodromal markers and the total LR were all higher in the converters (*P* < 0.001). In line with this, the converters had a higher posttest probability for prodromal PD than the control group. None of the converters or controls reached a posttest probability level of 50% or 80% for possible or probable PD, respectively.

In the total cohort (controls and converters), 25 subjects (7.8%) reached a probability of >5% (12 converters and 13 controls). In total, <10 subjects reached a probability of >10%, of whom the minority was a converter. In both groups, the maximum probability value that was reached was approximately 20%. Table 1 summarizes these findings.

**Table 1:** MDS research criteria in a group of converters vs. healthy controls

	Converters (*n* = 160)	HC (*n* = 320)	*P* value
Pretest probability	1.25 (0.75−2.0)^1^	1.25 (0.75−2.0)^2^	0.869
LR risk markers	0.96 (0.85−1.3)	0.85 (0.64−1.1)	<0.001
LR prodromal markers	0.77 (0.61−1.5)	0.61 (0.50−1.5)	<0.001
Total LR	0.81 (0.57−1.7)	0.67 (0.43−1.1)	<0.001
Posttest probability	1.29 (0.46−2.9)^1^	0.83 (0.39−1.8)^2^	0.014

All values are given as the median with the interquartile range between brackets. *HC* healthy control; *LR* likelihood ratio. 1: 35 below 50 years; 2: 67 below 50 years.

In the subgroup of converters (*n* = 73) with near-complete data and corresponding controls (*n* = 146), the presence of risk and prodromal markers was similar compared to the total group. T2DM and a positive family history were more common in the controls (*P* = 0.022 and *P* = 0.010, respectively). None of the prodromal markers were significantly more common in the converters (see Supplementary table 6*)*. The pretest probability in this group was also similar between converters and controls (Supplementary Table 7). The total LR and posttest probability was lower in the subgroup (*n* = 73) compared to the complete group of converters (*n* = 160). No significant differences in LR of risk and prodromal markers were found between converters and controls in this subgroup analysis. Table 2 shows the results of the criteria in the subgroups.

**Table 2:** MDS research criteria in a subgroup of converters with near complete data vs. healthy controls

	Converters (*n* = 73)	HC (*n* = 146)	*P* value
Pretest probability	1.25 (0.4−2.0)^1^	1.25 (0.4−2.0)^2^	0.988
LR risk markers	1.1 (0.85−1.2)	0.96 (0.75−1.2)	0.179
LR prodromal markers	0.61 (0.50−1.6)	0.63 (0.50−1.5)	0.615
Total LR	0.71 (0.52−1.7)	0.71 (0.51−1.2)	0.429
Posttest probability	0.87 (0.31−3.0)^1^	0.91 (0.41−1.9)^2^	0.991

All values are given as the median with the interquartile range between brackets. *HC* healthy control; *LR* likelihood ratio. 1: 18 below 50 years; 2:33 below 50 years.

### Sensitivity, specificity and positive predicted value (PPV)

None of the participants (controls and converters) reached the 50% or 80% probability threshold for probable prodromal PD. When lowering the level to 10%, the sensitivity was 0.6%, the PPV increased to 16.7%, and the specificity was 98.4%. At the 5% level, sensitivity reached 7.5%, the PPV 48%, while the specificity was 95.9%. The area under the curve (AUC) of the receiver operating characteristic (ROC) curve of the posttest probability was 0.577 (Supplementary Figure 2).

In the subgroup analysis (*n* = 73), the sensitivity was 1.4% at the 10% level, while the specificity was 97.2% and the PPV was 20%. At the 5% level, sensitivity was 6.8%, specificity 93.8% and the PPV 35.7%. The AUC-ROC of the overall posttest probability in this subgroup was 0.499.

### Likelihood ratio of single markers and age- and sex-based interactions

The positive likelihood ratio (LR+) and negative likelihood ratio (LR-) for prodromal PD was calculated for each marker in the full sample and for stratified age and sex groups (Supplementary Figure 3). The LR and their 95% CI were compared to the LR as defined by the MDS criteria. The LR+ for constipation, depression, and diabetes mellitus type 2 was lower compared to the MDS-defined LR+. For nonuse of caffeine, the LR+ for prodromal PD was higher in our cohort (3.6; 95% CI: 1.4−9.0) compared to that defined by the MDS (1.35).

We determined the interaction between each risk and prodromal marker and sex and age using logistic regression models. Only a significant interaction was found between constipation and age (*P* = 0.041) (Supplementary Table 8).

## Discussion

We applied the MDS research criteria for prodromal PD to a large cohort of individuals (*n* = 160) who developed PD during longitudinal follow-up in the Lifelines study. Our aim was to determine whether self-administered questionnaires, focusing on a subset of the risk and prodromal markers, could effectively distinguish PD-converters from controls. If successful, this method could enable cost-effective and large-scale identification of high-risk individuals. However, our results show that the available markers were insufficient to discriminate between converters and controls.

The incidence of PD in the Lifelines biobank can be considered to reflect the incidence of PD in the Northern parts of the Netherlands and was similar to a previous Dutch study *(*Supplementary figure 3*)*^14^. It was also similar to incidence rates found in other Western countries^15–17^. This suggests that the Lifelines cohort indeed represents the general population, which was an important prerequisite for the current study.

Using the twelve risk and prodromal markers available in Lifelines, it was theoretically possible to reach the 80% threshold for nearly all subjects (except females aged 50-54). However, none of the participants reached the 50% and 80% probability levels proposed by the MDS. In fact, the median probability for both converters and controls was very low (close to 1%). The low AUC of 0.577 further confirms the poor discriminative power of our approach. This was not attributable to missing data, as performance did not improve in a subgroup analysis of individuals with near-complete data. Instead, the low probability in converters was probably related to the fact that markers typically associated with a high LR (e.g., PSG-proven RBD or dopaminergic imaging) could not be assessed in Lifelines participants.

In addition, for each of the twelve available risk (except sex) and prodromal markers, we calculated the positive and negative likelihood ratios for prodromal PD in the Lifelines cohort. Overall, we found lower positive likelihood ratios than those reported in the MDS criteria, which is in line with a previous study. This was especially true for the prodromal markers of constipation and depression. Furthermore, we found that constipation significantly interacted with age, which indicates that age may impact the diagnostic accuracy of prodromal PD criteria, as indicated previously^18^. We did not find a significant effect of sex.

Our findings align with previous population-based studies, where a subset of the MDS criteria also demonstrated inadequate performance in identifying prodromal PD. The Hellenic Longitudinal Investigation of Aging and Diet (HELIAD) cohort showed that the MDS criteria yielded a median probability of 4.4% (IQR: 2.2 − 10.9) in a cohort of 961 community-dwelling individuals aged ≥65^13^. None of the 22 converters reached the 80% threshold, and only one reached the 50% threshold. Compared to our study, four additional markers were assessed, including subthreshold parkinsonism, erectile dysfunction, orthostatic hypotension (questionnaire) and pesticide exposure. Another population-based cohort (PRIPS) followed 715 subjects for five years^12^. None of the seven converters met the 80% threshold and only one met the 50% threshold. The criteria had a sensitivity of 14.3% and 43.0% at the 50% and 30% threshold, respectively, with a specificity of >98% for both thresholds. In the PRIPS study, four risk and four prodromal markers were used, including substantia nigra hyperechogenicity, subthreshold parkinsonism and hyposmia. These markers yield relatively high LRs, which could explain the higher sensitivity.

The highest sensitivity of the criteria was reported in the Bruneck study, a population-based cohort of 539 participants aged 55-94^10,11^. In the first three years of the study, there were six converters, of which four met the 80% threshold and all six met the 50% threshold. This resulted in a sensitivity of 67% and a specificity of 99% in the first three years. During a follow-up period of 10 years, 20 incident PD cases were found, and the sensitivity decreased to 54.6% and 11.1%, at five- and ten-year follow-up, respectively. Nearly all markers were available, except for PSG-proven RBD, dopaminergic imaging and genetic screening. The large availability of markers likely contributed to these high scores.

The accuracy of the criteria improves when applied in enriched cohorts. For instance, in a cohort of 121 subjects with idiopathic RBD (iRBD), the criteria were found to have a sensitivity of 81.4% and a specificity of 67.9% for conversion to PD in the next 4 years^9^. Both increased with longer follow-up duration. Specificity and positive predictive value were 100% after 10-year follow-up. In other words, all converters met the 80% threshold of the criteria before conversion. Similar results were found in another iRBD cohort and in an LRRK2-carrier cohort^7,8^. The presence of preselected markers thus strongly influences the accuracy of the criteria.

Based on the collective findings from earlier studies and the results presented here, it is apparent that the MDS criteria are not well-suited for screening for prodromal PD in the general population. However, they prove effective in detecting prodromal PD within enriched cohorts, where individuals are pre-selected based on their elevated risk for developing PD. To some extent, this is circular reasoning. In iRBD-cohorts a substantial majority (>80%) will eventually develop PD or dementia with Lewy bodies^19^. In contrast, in the general population, around 1% will develop PD, and the majority of these cases (60–70%) may not even experience prodromal symptoms^2^. Consequently, the MDS research criteria may fail to detect a significant proportion of individuals at risk. Accurate detection of at-risk individuals is mainly dependent on ancillary testing, which is impractical on a larger scale within the context of a biobank such as Lifelines. However, there are opportunities to enhance the identification of high-risk individuals in a population-based cohort. One strategy could be to incorporate a smell test, online motor testing and standardized questionnaires on prodromal symptoms.

The PREDICT-PD algorithm provides an alternative to the MDS prodromal criteria for estimating PD risk, using only remotely assessable markers^20^. These include a keyboard tapping task and at-home olfaction testing. The PREDICT-PD criteria are similar to the MDS criteria, with minor differences. Notably, PREDICT-PD employs continuous variables for age, motor impairment, and olfaction, compared to MDS’s categorical age intervals and dichotomous variables. In addition, PREDICT-PD includes some risk markers, such as head injury and use of alcohol, which are not included in the MDS criteria. In PREDICT-PD, a cohort of 1323 participants aged 60–80 were followed for six years, during which 10 subjects converted to PD. The PREDICT-PD model performed similarly to the MDS criteria^21^. Ongoing longitudinal follow-up of the PREDICT-PD cohort aims to refine PD risk estimation.

In the current study, we only evaluated data from questionnaires. However, Lifelines also offers analysis of (previously) collected biosamples, including blood and fecal samples, and genetic material. The analysis of these samples fell beyond the scope of this research but can potentially be valuable in the risk assessment of developing PD. Uric acid and T2DM, both markers based on biological assessments, are already included in the MDS criteria. Contrary to recent studies linking T2DM to an elevated risk of PD^22^, we found a lower prevalence of T2DM in PD-converters compared to controls (4% vs 10%). In a cohort of approximately 40,000 Lifelines participants aged 50-70 years, the frequency of a self-reported diagnosis of diabetes was 5%. This suggests that the relatively high frequency of T2DM in our age- and sex-matched control cohort (*n* = 320) could be attributed to a selection bias. Moreover, other biological markers associated with PD, such as cholesterol levels or inflammatory markers, could improve the risk estimation^23,24^. Another promising candidate for screening for PD is a seeding amplification assay (SAA) of pathological alpha-synuclein^25^, which has recently become available for serum samples and may be a very promising candidate to screen for the risk of PD in the general population^26^.

The most important limitation of this study is that a PD diagnosis in Lifelines participants was self-reported and could not be verified by a neurologist. It is therefore possible that some of the 160 subjects indicated as converters in fact had a different diagnosis (i.e. atypical parkinsonism, other forms of tremor or extrapyramidal side-effects of medication). Due to the design of the Lifelines biobank, we could not verify this self-reported diagnosis, nor did we have access to accurate records of medication use. Taking this into consideration, the incidence of PD in our cohort was in line with previous reports, which may suggest that this bias is limited.

Moreover, we could not apply the full spectrum of risk and prodromal markers of the MDS criteria, because many of these markers are not available in a broad population-based cohort, which did not focus on PD specifically. We were able to evaluate the following prodromal markers: possible RBD, constipation, excessive daytime somnolence, urinary dysfunction, depression and global cognitive deficit. Excessive daytime sleepiness was properly evaluated with the Epworth Sleepiness Scale and cognitive deficit was properly evaluated with a Mini-Mental State examination. However, the other markers were not assessed using established questionnaires. To assess constipation, we combined a single question from the ROME-III questionnaire (“how often did you have <3 bowel movements a week in the past three months?”) with information on laxative use. For urinary incontinence or depression, simple yes/no questions were used. Finally, for RBD, a single question from the Pittsburgh Sleeping Quality Index was used. The responses may therefore have been unspecific. Additionally, the more objective prodromal markers from the MDS criteria could not be assessed. These include PSG-confirmed RBD, motor testing, olfaction testing, dopaminergic imaging and orthostatic hypotension and are associated with higher LRs. Furthermore, data were missing for several of the assessed markers. However, in a subgroup analysis of 73 converters with near-complete data, the performance of the criteria did not improve, suggesting that this had a limited impact.

In conclusion, we studied the largest population-based prodromal PD cohort to date using data from questionnaires. We could not identify PD-converters using a limited set of the MDS prodromal PD criteria in our Lifelines cohort. An important explanation for this result was that many data were not available for the complete MDS-defined set of prodromal and risk factors. Particularly those that depend on ancillary testing and are associated with high likelihood ratios were not available. Our study, as well as previous population-based studies, illustrate that detection of prodromal PD from a general population is challenging, using the prodromal MDS research criteria in its current form. Therefore, the development of objective markers that are easy to apply for accurate identification of prodromal PD in large general cohorts is urgently needed.

## Methods

### Subjects

The subjects of this study are all participants of Lifelines (www.lifelines.nl). Lifelines is a multi-disciplinary prospective population-based cohort study examining in a unique three-generation design the health and health-related behaviors of 167,729 persons living in the North of the Netherlands. It employs a broad range of investigative procedures in assessing the biomedical, socio-demographic, behavioral, physical and psychological factors, which contribute to the health and disease of the general population, with a special focus on multi-morbidity and complex genetics^27^. At each assessment, participants indicate whether they have received a new diagnosis.

We selected 174 subjects from the Lifelines database, who self-reported to have developed PD after their first assessment. Out of these, 14 were excluded for the following reasons: antiparkinsonian medication use at baseline, a reported stroke before the reported diagnosis of PD, or not attending the first assessment, but a reported diagnosis of PD in the first questionnaire. This resulted in a group of 160 cases, who converted to PD during follow-up in lifelines (‘PD-converters’). Every converter was randomly matched to two age- and sex-matched controls. Informed consent was signed by all participants at the start of Lifelines. Due to privacy regulations of Lifelines and possible identification of individuals, small numbers (<10) are written as ‘<10 subjects’. The Lifelines protocol was approved by the medical ethical committee (METc) of the University Medical Center Groningen (UMCG) under number 2007/152.

### Data collection

Data in Lifelines is collected via questionnaires and physical visits. Assessment rounds are repeated every five years and consist of two alternating visits and two extensive questionnaires. During the visits, biomaterials are collected (e.g., blood and urine), physical measurements are performed (e.g., blood pressure, ECG, spirometry) and supervised questionnaires are collected (e.g., Mini Mental State Examination (MMSE)). Different questionnaires are sent out in between the assessment rounds. Questionnaires or visits receive different codes. For example, 1AQ1 indicates the first questionnaire in the first assessment round, and 3AV2 reflects the second visit in the third assessment round. Questionnaires that are sent out in between the rounds are denoted as 1B, 1C or 2B.

### Incidence

The incidence (number of new cases per 1,000 person-years) of PD in the lifelines cohort was calculated according to the following formula [1]:

1 $${\rm{Incidence}}={({\rm{new}}\,{\rm{cases}})}/{({\rm{time}}\,{\rm{at}}\,{\rm{risk}})}\,*\,{1,000}$$

The time at risk is defined as the period during which a subject is followed but does not have PD. The follow-up started at inclusion in Lifelines and ended when a subject reported to have developed PD, deceased, or was lost to follow-up. The incidence was calculated for each age category, arbitrarily set at <45; 45–54; 55–64; 65–74; 75–84 and >85. This was calculated for the group as a whole and for men and women separately.

### Prodromal and risk markers

The prodromal and risk markers were assessed using questionnaires collected before the reported moment of conversion. Questionnaires collected at or after the assessment in which a diagnosis of PD was reported were not considered. For controls, all questionnaires were considered. Lifelines did not include a questionnaire specific for prodromal PD. Thus, markers were inferred from other questionnaires. Not all markers were available, and in some cases suboptimal proxies were used. Furthermore, within one subject the questionnaires were not collected at the same timepoint. For instance, urinary dysfunction was assessed at 1AQ1, whereas excessive daytime sleepiness was assessed approximately 2 years later at 1C. The calculated probability for prodromal PD in this study is therefore not a reflection of one moment in time, but a combination of different time points.

The six available risk markers included male sex, non-smoking, physical inactivity, nonuse of caffeine, family history of PD and T2DM. The six available prodromal markers included constipation, urinary dysfunction, possible RBD, excessive daytime somnolence (EDS), depression and global cognitive impairment.

The age and sex of all participants (converters and controls) were determined at the first assessment. The following five markers were assessed only once: positive family history, physical inactivity, possible RBD, excessive daytime somnolence and urinary dysfunction. The other six markers (smoking status, caffeine use, diabetes mellitus type 2, constipation, depression and global cognitive deficit) were assessed multiple times during follow-up. These markers were scored negative if all available assessments were negative. They were scored positive if one or more assessments were positive. For instance, if a subject was not constipated at assessment 2 A, but was constipated at 3 A, that person was scored as constipated. If it was the other way around (constipated at 2 A but not at 3 A), that person was also scored as constipated.

The presence of most of these markers was based on self-administered questionnaires. Some questionnaires were specifically developed for the topic (e.g., Epworth Sleepiness Scale (ESS) for EDS), whereas other markers were assessed using questionnaires not specific to the topic^28^. For instance, a questionnaire for RBD was not included. As a proxy, we used a question from the Pittsburgh Sleeping Quality Index: “if you have a roommate or bed partner, ask him/her how often in the past month during your sleep you had twitching or kicking of the legs”^29^. Global cognitive deficit was assessed with the MMSE in subjects aged 65 or older^30^. To assess constipation, we analyzed which subjects used laxatives, and combined this information with the answers on a question of the ROME III questionnaire^31^. Table 3 shows how markers were assessed and when this was done. Supplementary Table 9 gives an overview of all of the aspects of the MDS research criteria and how these were assessed in the current study. Considering the limitations outlined above, the highest achievable posttest probability that could have been theoretically reached in a male aged 80 or older would be 98%. Females between 50 and 54 cannot reach the 80% threshold, but for the other age categories, it was theoretically possible to reach 80% for both males and females.

**Table 3:** Assessed prodromal and risk markers in Lifelines

Assessed marker:	Assessed how:	Scored if:	Assessed in:
Presence of Parkinson’s disease	Parkinson’s disease / Could you indicate which of the following disorders you have (had)?	Yes	1A, 1B, 1C, 2A, 3A, 3B
	Parkinson’s disease / Did the health problems listed below start since the last time you filled in the Lifelines questionnaire?		
Smoking status	Current smoker (yes/no)	Current/former/never yes	1A, 1B, 1C, 2A, 2B, 3A
	Never smoker (yes/no)		
	Ever smoker (yes/no)		
Use of caffeine	How often did you drink coffee in the past month? Include instant coffee and decaf / score range from 1 (not this month) through 7 (6-7 days a week)	Value 5 or higher (2-3 days a week or more)	1B, 1C or 2A
	Which type of coffee did you drink in the past month? Caffeinated coffee / score range from 1 (never) through 4 (always)	Value 3 (often) or higher	
First-degree relative with Parkinson’s disease	Parkinson’s disease / Do/did your biological children/father/mother/ (half)siblings have any of the following conditions (yes/no)	Yes	1B
Diabetes mellitus type 2	Do you have diabetes? What type?	Yes, type 2	1A, 1B, 1C, 2A, 3A
	Type 2 diabetes / did the health problems listed below start since the last time you filled in the lifelines questionnaire		
Physical inactivity	SQUASH: minutes per week moderate/vigorous intensity activity^32^	<60 minutes per week	1A
Constipation	ROME III questionnaire: how often did you have fewer than 3 (0-2) bowel movements a week in the past 3 months? Score range from 1 (rarely or never) - 5 (always)^31^	Value 2 (sometimes) or higher	2A, 3A
	Use of prescribed laxatives - ATC codes	Yes	1A
	Use of over-the-counter laxatives	Yes	1A, 1B, 1C, 2A, 2B
Possible RBD	PSQI: if you have a roommate or bed partner, ask him/her how often in the past month you had twitching or kicking of the legs^29^	Value 3 (once or twice per week) or higher	1B
Excessive daytime somnolence	Epworth Sleepiness Scale^28^	Total score ≥ 11	1C
Urinary dysfunction	Incontinence / could you indicate which of the following disorders you have (had)?	Yes	1A
Depression	Depression / could you indicate which of the following disorders you have (had)?	Yes	1A, 1B, 1C, 2A, 3A
	Depression / did the health problems listed below start since the last time you filled in the Lifelines questionnaire?		
Global cognitive deficit	MMSE in >65 years old^30^	Score <24	1A, 3A

*SQUASH* Short Questionnaire to Assess Health-enhancing physical activity, *ATC code* Anatomical Therapeutic Chemical code, *RBD* REM-sleep behavior disorder, *PSQI* Pittsburgh Sleep Quality Index, *MMSE* Mini-Mental State Exam.

### Calculation posttest probability for prodromal PD using the MDS criteria

The MDS research criteria^5,6^ for prodromal PD include likelihood ratios (LR) of the risk and prodromal markers. These are multiplied to reach a total LR. When a marker was not assessed, the LR of that marker was set to 1. To calculate the eventual posttest probability, expressed as a percentage, the following formulas were used [2, 3, 4]:

2 $${\rm{pretest}}\,{\rm{odds}}={\rm{pretest}}\,{\rm{probability}}/{(1-{\rm{pretest}}\,{\rm{probability}})}$$

3 $${\rm{Posttest}}\,{\rm{odds}}={\rm{pretest}}\,{\rm{odds}}\,*\,{\rm{total}}\,{\rm{LR}}$$

4 $${\rm{Posttest}}\, {\rm{probability}}={\rm{posttest}}\,{\rm{odds}}/{(1+{\rm{posttest}}\,{\rm{odds}})}$$

The pretest probability is indicated by the MDS criteria and is based on the age of the subject. The MDS provided a pretest probability for age categories starting from the age of 50. For ages below 50, no pretest probability is provided. The posttest probability was calculated for each participant separately. We calculated the outcome of the research criteria twice. First, we calculated it for the whole group (i.e., all converters vs. all controls). Second, we repeated the calculation for a subgroup of 73 subjects with near-complete data. Participants were selected in the near-complete subgroup if less than three markers were missing. This subgroup was matched for sex and age with 146 controls.

### Statistical analysis

The difference in the presence of the separate risk and prodromal markers between the groups was assessed with a Chi-square test, or a Fisher’s exact test depending on the characteristics of the data. We tested the total LR and the posttest probability for normality with a Q-Q plot and a Kolmogorov-Smirnov test. The total LR and the posttest probability were compared between the groups with a Mann-Whitney U test. Sensitivity, specificity and positive predictive value were calculated for the 80%, 50%, 10% and 5% level for probability for prodromal PD. Sex- and age-associated interactions with the single risk and prodromal markers were determined with logistic regression. The dependent variable was converter/nonconverter. Independent variables were defined as the marker (category), age (continuous in years), sex (male/female), and the interaction terms marker*age and marker*sex. The statistical analyses were performed in SPSS, version 28.

### Supplementary information

Supplemental material

## Data Availability

Data may be obtained from a third party and are not publicly available. Researchers can apply to use the Lifelines data used in this study. More information about how to request Lifelines data and the conditions of use can be found on their website (https://www.lifelines.nl/researcher/how-to-apply/).
