# Supplementary material for: A retrospective study of the MDS criteria for prodromal Parkinson’s disease in the general population
Source: NPJ Parkinsons Dis. 2024 Jun 26;10:125. doi: 10.1038/s41531-024-00739-6 (PMC11208573; doi:10.1038/s41531-024-00739-6)
Supplement: Supplementary file 1 — Supplemental material [file 41531_2024_739_MOESM1_ESM.pdf]

Supplementary table 1: Demographics of converters and HC

	Converters (n=160)	HC (n=320)	P-value
**Sex, male (%)**	100 (63%)	188 (59%)	0.429
**Age baseline**	59 (11)	59 (11)	0.979
**Age reported diagnosis**	64 (10)	NA	NA
**Duration of follow up***	5.6 (4.0)	7.1 (4.1)	<0.001

Values are given as the mean with the standard deviation between brackets. HC: healthy control; FU: follow-up; NA: not applicable. * For the converter group, follow-up ended at the moment the diagnosis of PD was reported; for the HC group, FU ended at the last measurement.

Supplementary table 2: Subgroup analysis with near-complete data: Demographics of subgroup of converters and controls

	CONVERTERS (N=73)	HC (N=146)	P-VALUE
**SEX, MALE (%)**	52 (71%)	102 (71%)	1
**AGE BASELINE**	57 (11)	58 (11)	0.425
**AGE REPORTED DIAGNOSIS**	66 (9.0)	NA	NA
**DURATION FU**	8.7 (3.1)	6.9 (4.1)	0.001

Values are given as the mean with the standard deviation between brackets. HC: healthy control; FU: follow-up; NA: not applicable.

Supplementary table 3. Incidence rates of PD for men, women and all cases

	Men			Women			All		
Age group	N	Person-years	IR (95% CI)	N	Person-years	IR (95% CI)	N	Person-years	IR (95% CI)
<45	<10	$1.4*10^5$	0.028 (0.01-0.08)	<10	$2.2*10^5$	0.027 (0.01–0.06)	10	362532	0.028 (0.01-0.5)
45-54	36	99522	0.36 (0.26-0.50)	19	147043	0.13 (0.08-0.20)	55	246660	0.22 (0.17- 0.29)
55-64	31	52096	0.60 (0.41-0.85)	13	69307	0.19 (0.11-0.32)	44	121428	0.36 (0.27-0.49)
65-74	19	24271	0.78 (0.50-1.2)	17	24845	0.68 (0.43-1.1)	36	49123	0.73 (0.53-1.02)
75-84	<10	3066	2.9 (1.5-5.6)	<10	$2.9*10^3$	1.4 (0.52–3.7)	13	5937	2.2 (1.3-3.7)
>85	<10	$1.2*10^2$	8.1 (1.1-57)	<10	$1.6*10^2$	6.3 (0.89-45)	<10	$2.8*10^2$	7.1 (1.8-28)
Total	100	319908	0.31 (0.26–0.38)	60	465754	0.13 (0.10-0.17)	160	785962	0.20 (0.17-0.24)

IR (95% CI): incidence rate with 95% confidence interval. Due to privacy policies of Lifelines, groups smaller than 10 are denoted as ''<10'' and the accompanying person-years is denoted as a power of ten.

Supplementary table 4. Presence of risk and prodromal markers in converters vs. controls

Marker	PD-converters			Controls			P-value
	Yes, %	No, %	Missing, N	Yes, %	No, %	Missing, N	
**Risk markers**							
Male sex	62.5%	37.5%	0	59%	41%	0	0.429
Smoking status	10%*	48%**<br>42%**	<10	14%*	52%**<br>34%***	<10	0.172
Nonuse caffeine	12%	88%	87	3%	97%	<90	0.004
Physical inactivity	18%	82%	23	14%	86%	47	0.211
First-degree relative with PD	13%	87%	13	4%	96%	52	<0.001
Diabetes mellitus type 2	4%	96%	<10	10%	90%	<10	0.018
**Prodromal markers**							
Constipation	34%	66%	92	27%	73%	86	0.299
Possible RBD	16%	84%	67	6%	94%	87	0.006
Excessive daytime somnolence	15%	85%	81	8%	92%	93	0.084
Urinary dysfunction	3%	97%	<15	3%	97%	19	0.975
Depression	9%	91%	0	11%	89%	0	0.597
Global cognitive deficit	5%	95%	>100	8%	92%	189	0.758

* : Current smoker; **: former smoker; ***: never smoker. PD: Parkinson's disease, RBD: REM-sleep behavior disorder. When calculating the percentages for ''yes'' and ''no'', we treated the sum of ''yes'' and ''no'' cases as 100%. Missing cases had no impact on the ''yes'' and ''no'' percentages. Due to privacy policies of Lifelines, groups smaller than 10 are denoted as ''<10''.

Supplementary table 5. Age and pretest probability for converters vs. Controls

Age	Pretest probabilility, %	Converters	Controls
<50	Not applicable	35	67
50-54	0.4%	30	54
55-59	0.75%	19	42
60-64	1.25%	25	53
65-69	2.0%	25	51
70-74	2.5%	23	49
75-79	3.5%	<20	<20
>79	4%	<10	<10

Due to privacy policies of Lifelines, groups smaller than 10 are denoted as ''<10''.

Supplementary table 6. Subgroup analysis with near-complete data: Presence of risk and prodromal markers in the subgroup of converters versus controls

**Marker**	**PD-converters**			**Controls**			**P-value**
	**Yes, %**	**No, %**	**Missing, N**	**Yes, %**	**No, %**	**Missing, N**	
**Risk markers**							
Male sex	71%	29%	0	71%	29%	0	1
Smoking status	7%*	48%**<br>45%***	0	11%*	52%**<br>37%***	<10	0.391
Nonuse caffeine	13%	87%	<15	5%	95%	<45	0.055
Physical inactivity	13%	87%	<10	12.5%	87.5%	26	0.972
First-degree relative with PD	12.5%	87.5%	<10	2.5%	97.5%	<30	0.010
Diabetes mellitus type 2	1%	99%	<10	10%	90%	<10	0.022
**Prodromal markers**							
Constipation	22%	78%	17	25%	75%	40	0.567
Possible RBD	17%	83%	<10	8%	92%	<40	0.094
Excessive daytime somnolence	14%	86%	<10	7%	93%	<45	0.218
Urinary dysfunction	3%	97%	<10	4%	96%	<10	1
Depression	10%	90%	<10	12%	88%	0	0.646
Global cognitive deficit	0%	100%	53	11%	89%	<100	0.120

* : Current smoker; **: former smoker; ***: non- smoker. PD: Parkinson's disease, RBD: REM-sleep behavior disorder. When calculating the percentages for ''yes'' and ''no'', we treated the sum of ''yes'' and ''no'' cases as 100%. Missing cases had no impact on the ''yes'' and ''no'' percentages. Due to privacy policies of Lifelines, groups smaller than 10 are denoted as ''<10''.

Supplementary table 7. Subgroup analysis with near-complete data: age and pretest probability of the subgroup of converters versus controls.

Age	Pretest probabilility, %	Converters	Controls
<50	Not applicable	18	33
50-54	0.4%	17	29
55-59	0.75%	<10	19
60-64	1.25%	13	23
65-69	2.0%	<10	21
70-74	2.5%	<10	<10
75-79	3.5%	<10	<10
>79	4%	<10	<10

Due to privacy policies of Lifelines, groups smaller than 10 are denoted as ''<10''.

Supplementary figure 1. ROC curves of the posttest probability

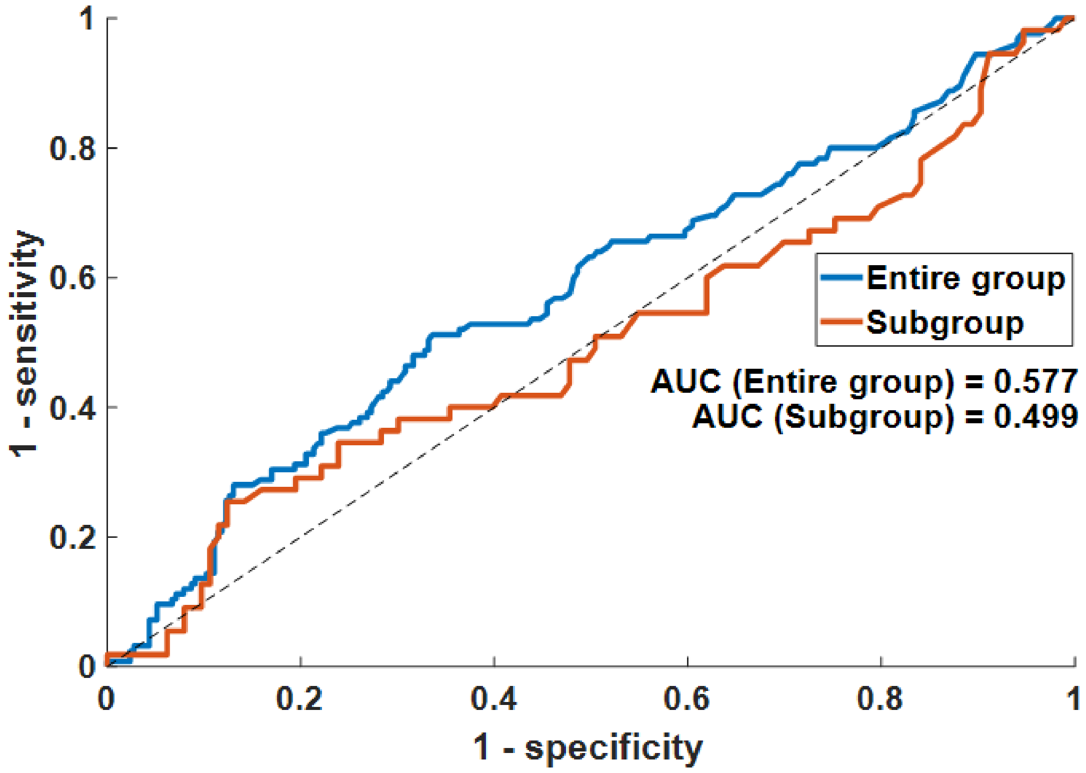

ROC curve of posttest probability between PD-converters and controls in the entire group (AUC = 0.577) and the ROC curve of the subgroup of 73 converters vs. 146 controls with near-complete data. ROC: receiver operating characteristic; AUC: area under curve.

Supplementary figure 2. Incidence of PD in Lifelines vs. previous study in the Netherlands (Rotterdam study).

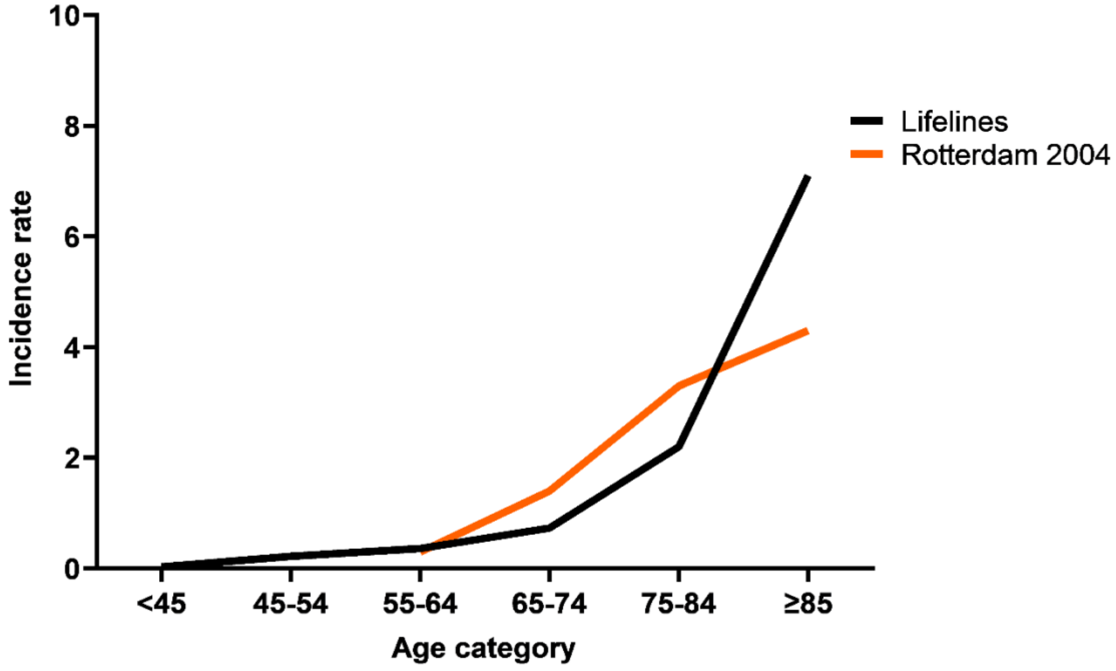

Incidence rate of PD in Lifelines for men and women combined compared to a previous Dutch study performed in 2004 by De Lau et al. (14).

Supplementary Figure 3. Likelihood ratio of risk and prodromal markers

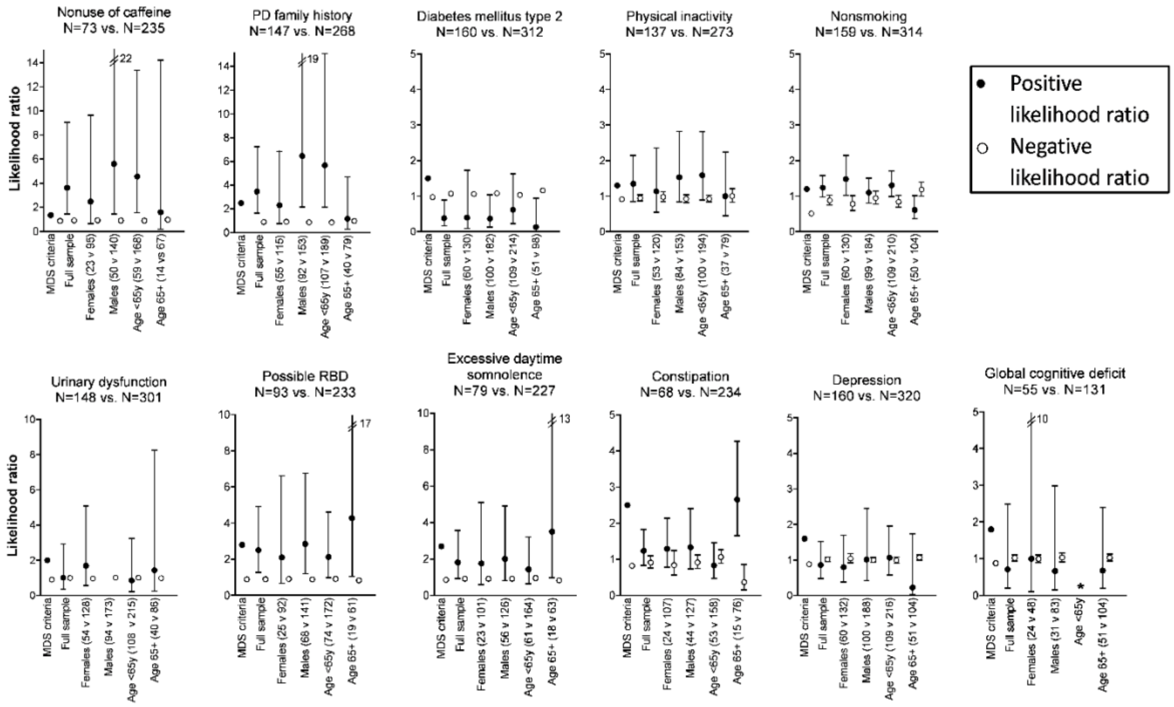

Positive and negative likelihood ratio for the risk and prodromal markers. These were calculated for the full sample and in subsamples stratified by age (<65 and 65+) and sex (male/female). The number (N) below the marker is the number of converters vs. controls used to calculate the LRs. The numbers in brackets in the description on the X-axes are the number of converters vs. controls. Error bars indicate the 95% confidence interval. In the analysis of smoking, we grouped subjects as non-smokers if they never smoked, and as smokers if they were current smokers or ex-smokers. *: global cognitive deficit was assessed with the Mini-mental state exam, which was only measured in those who are 65 years or older. PD: Parkinson's disease; RBD: REM-sleep behavior disorder.

Supplementary table 8. Logistic regression coefficients.

	Marker B ± S.E. (p-value)	Age B ± S.E. (p-value)	Sex B ± S.E. (p-value)	Marker*age B ± S.E. (p-value)	Marker*sex B ± S.E. (p-value)	Constant B ± S.E. (p-value)
Nonsmoking	8.7 ± 5.9<br>0.141	0.1 ± 0.1<br>0.34	3.0 ± 2.3<br>0.184	-0.1 ± 0.1<br>0.34	-2.7 ± 2.1<br>0.2	-10.1 ± 6.4<br>0.115
nonuse caffeine	-1.0 ± 2.9<br>0.721	-0.9 ± 0.1<br>0.434	-1.6 ± 2.3<br>0.489	0.085 ± 0.1<br>0.479	5.5 ± 6.1<br>0.371	-0.3 ± 6.4<br>0.962
Physical inactivity	-0.9 ± 1.6<br>0.593	-0.04 ± 0.1<br>0.558	0.9 ± 1.5<br>0.546	0.04 ± 0.1<br>0.551	3.0 ± 4.0<br>0.446	-0.7 ± 4.2<br>0.868
First-degree relative PD	-0.2 ± 2.3<br>0.919	0.04 ± 0.03<br>0.233	-0.3 ± 0.7<br>0.702	-0.04 ± 0.02<br>0.201	1.1 ± 1.5<br>0.475	**-4.0 ± 2.1**<br>**0.049**
Diabetes mellitus type 2	0.4 ± 2.8<br>0.896	0.2 ± 0.1<br>0.1	1.0 ± 1.9<br>0.589	-0.2 ± 0.1<br>0.11	-2.2 ± 4.8<br>0.653	-8.4 ± 6.1<br>0.169
Constipation	-3.0 ± 1.7<br>0.087	**-0.04 ± 0.02**<br>**0.015**	0.5 ± 0.4<br>0.158	**0.06 ± 0.03**<br>**0.041**	-0.1 ± 0.6<br>0.84	0.7 ± 1.0<br>0.491
Possible RBD	0.1 ± 1.2<br>0.919	-0.02 ± 0.03<br>0.375	0.4 ± 0.6<br>0.468	0.02 ± 0.04<br>0.655	0.4 ± 0.8<br>0.7	-0.4 ± 1.6<br>0.778
EDS	-4.0 ± 2.8<br>0.149	-0.01 ± 0.01<br>0.326	**0.7 ± 0.3**<br>**0.03**	0.08 ± 0.05<br>0.081	-0.09 ± 0.9<br>0.921	-0.7 ± 0.9<br>0.396
Urinary dysfunction	1.0 ± 3.8<br>0.795	0 ± 0.01<br>0.977	0.3 ± 0.2<br>0.143	-0.01 ± 0.06<br>0.911	-21.2 ± 23193<br>0.999	-0.9 ± 0.6<br>0.135
Depression	2.2 ± 2.2<br>0.319	0.0 ± 0.01<br>0.81	0.1 ± 0.2<br>0.576	-0.04 ± 0.04<br>0.259	0.2 ± 0,7<br>0.74	-0.9 ± 0.6<br>0.128
Global cognitive deficit	7.0 ± 6.7<br>0.302	0.05 ± 0.03<br>0.054	-0.2 ± 0.3<br>0.543	-0.1 ± 0.1<br>0.301	-0.4 ± 1.5<br>0.786	**-4.1 ± 1.8**<br>**0.022**

Significant values (P<0.05) are indicated in bold. B=regression coefficient; S.E.= standard error; PD, Parkinson’s disease; RBD, REM-sleep behavior disorder; EDS: excessive daytime somnolence

Supplementary table 9. Overview of markers available in Lifelines and their likelihood ratios (LR)

	**Marker**	**LR if positive**	**LR if negative**
**Available in Lifelines**			
**Risk markers**	Male sex	1.2 (male)	0.8 (female)
	Nonuse of caffeine	1.35	0.88
	Nonsmoking		
	Current smoker	NA	0.51
	Never smoker	1.2	NA
	Former smoker	NA	0.91
	First-degree relative with PD	2.5	NA
	Diabetes mellitus (type II)	1.5	0.97
	Physical inactivity	1.3	0.91
**Prodromal markers**	Possible RBD (proxy from questionnaire)	2.8	0.89
	Constipation	2.5	0.82
	Excessive daytime somnolence	2.7	0.86
	Urinary dysfunction	2.0	0.90
	Depression (± anxiety)	1.6	0.88
	Global cognitive deficit	1.8	0.88
**Not available in Lifelines**			
**Risk markers**	Regular pesticide exposure	1.5	NA
	Occupational solvent exposure	1.5	NA
	Known gene mutation (with intermediate-strength penetrance)	*	NA
	Polygenic risk score	1.57	0.45
	SN hyperechogenicity	3.4	0.38
	Low plasma urate levels	1.8 (in men)	0.88 (in men)
**Prodromal markers**	PSG-proven RBD	130	0.65
	Dopaminergic PET/SPECT clearly abnormal (eg, <65% normal, 2 SDs below mean)	43.3	0.66
	Subthreshold parkinsonism (UPDRS-III >3 excluding action tremor or MDS-UPDRS-III >6 excluding postural and action tremor)	9.6	0.55
	Abnormal quantitative motor testing	3.5	0.6
	Olfactory loss	6.4	0.40
	Orthostatic hypotension (OH) – neurogenic OH	18.5	0.88
	Symptomatic OH	3.2	0.80

	Erectile dysfunction	3.4 (in men)	0.87 (in men)

NA: not applicable; PD: Parkinson's disease; RBD: REM-sleep behavior disorder; SN: substantia nigra; PSG: polysomnography: PET/SPECT: positron emission tomography/ single-photon emission computed tomography; SD: standard deviation: MDS-UPDRS: movement disorder society unified Parkinson's disease rating scale. *: dependent on age-related penetrance, for details, see updated paper on MDS criteria (6).

With the available risk and prodromal markers in Lifelines, the highest achievable likelihood ratio (LR) is 1032. This is sufficient to reach the 80% threshold proposed by the movement disorder society for probable prodromal PD for males in all age categories. For example, the minimum LR to reach 80% in a person aged 50-54 is 1000, whereas it is 95 in a person of 80 or older. Below are two examples with the data that was available in Lifelines.

1. Example: A 70-year-old female with diabetes mellitus type 2, who never smoked and drinks coffee. She has constipation and depression, but no daytime somnolence. She has a prior probability of 2.5% because of her age. She has a total LR of 0.8 (female) * 1.5 (diabetes mellitus type 2) * 1.2 (nonsmoking) * 0.88 (caffeine) * 2.5 (constipation) * 1.6 (depression) * 0.86 (no daytime somnolence) = 4.4.
    a. With an LR of 4.4 and a pretest probability of 2.5, this person has a 10% chance of having prodromal PD. To reach the 80% threshold at 70-years-old a LR of 155 is needed. Thus, this person does not meet the criteria for prodromal PD.
2. Example: An 85-year-old male who used to smoke and drinks no coffee has a brother with PD. He is physically inactive and excessively sleepy during the day. According to his wife he kicks his legs during his sleep almost every night. He is also constipated. He is not incontinent, and his cognition is normal. He has a prior probability of 4%. He has a total LR of 1.2 (male) * 0.91 (former smoker) * 1.35 (no caffeine) * 2.5 (brother with PD) * 1.3 (physical inactivity) * 2.7 (excessive daytime somnolence) * 2.8 (possible RBD) * 2.5 (constipation) * 0.9 (no incontinence) * 0.88 (normal cognition) = 72.
    a. With an LR of 72 and a pretest probability of 4%, this person has a 75% chance of having prodromal PD. To reach the 80% threshold at 85-years-old an LR of 95 is needed. This person therefore does not meet the criteria of prodromal PD. However, he did reach the 50% threshold of possible prodromal PD.
